# Supplementary material for: Iron-mediated organic matter decomposition in humid soils can counteract protection
Source: Nat Commun. 2020 May 7;11:2255. doi: 10.1038/s41467-020-16071-5 (PMC7206102; doi:10.1038/s41467-020-16071-5)
Supplement: Supplementary file 1 — Supplementary Information [file 41467_2020_16071_MOESM1_ESM.pdf]

Supplementary Information for

Iron-mediated organic matter decomposition in humid soils can counteract protection

By Chen et al.

Supplementary Methods

Preparation of ^{13}C-labeled plant-derived DOM

^{13}C-DOM was extracted from ^{13}C-labeled bermudagrass. A pulse-labeling method was used to label "Tifton-85" bermudagrass (*Cynodon dactylon x Cynodon nlemfuencis*) with $^{13}CO_2$ (99.999% atom, Cambridge Isotope Laboratories Inc). Before labeling, bermudagrass were grown in flats of C-free sand with some residual potting medium under greenhouse conditions for four weeks. All flats were fertilized with 0.7 mmol $(^{15}NH_4)_2SO_4$ once a week. For ^{13}C pulse labeling, bermudagrass was transported to a Conviron growth chamber with temperature of 35°C and CO_2 concentration of 500 ppm maintained using a Qubit G400-2 gas mixing system coupled with a S157 infrared CO_2 analyzer (Kingston, Ontario, CA). Pulse labeling was carried out once a week for 6 weeks. Bermudagrass were exposed to $^{13}CO_2$ for 8 h with a flow rate of $^{13}CO_2$ approximately 0.5 L/min. At the end of labeling, aboveground biomass was harvested, immediately frozen, freeze-dried, and then ground using a Wiley-mill to <1 mm. DOM extractions were conducted in a shaker at 140 rpm for 2 days with a solid-to-water ratio of 1:5, followed by centrifugation. The supernatant was filtered through a 0.2 µm membrane filter. The derived DOM solution had 10.3 % ^{13}C.

Water-extractable native soil organic matter (SOM)

Water-extractable native SOM was obtained by mixing 20 g of the field soils with 45 mL high purity water (Keiluweit et al. 2017). The suspension was mixed and agitated at 100 rpm for 1 h in the dark and centrifuged at 14,000 g for 10 min. The supernatant was subsequently filtered through 0.2-µm nylon membranes. Extracted OC was quantified using a Shimadzu TOC analyzer.

Fe isotope analysis by ICP-MS

Samples analyzed by ICP-MS were measured in an optimized DRC mode using reactive NH_3 gas to minimize potential interferences from Ar, O, and H containing complexes that could convolute the mass-to-charge signal of Fe isotopes. All results were referenced to ^{54}Fe with correction of chromium concentrations by monitoring ^{52}Cr. The accuracy of this method was tested with Fe isotopic reference material IRMM-014 over multiple days (n=3). Repetitive measurements of isotopic fractions in IRMM-014 were stable (4.56±0.11%, 93.12±0.22%, 2.02±0.12%, and 0.29±0.02% for $f^{54}Fe$, $f^{56}Fe$, $f^{57}Fe$ and $f^{58}Fe$, respectively, n = 90). The validity of Fe isotope measurements was also tested using mixed solutions of known isotopic ratios. Linear regressions between measured and calculated isotopic ratios of the mixed solution showed R^2 values of 0.982, 0.987, 0.992, and 0.971 for $f^{54}Fe$, $f^{56}Fe$, $f^{57}Fe$ and $f^{58}Fe$, respectively (n = 87).

ESI FTICR-MS sample preparation, analysis and data interpretation

Fourier transform ion cyclotron resonance mass spectrometry (FTICR-MS) analysis was used to characterize the molecular composition of ^{13}C-DOM and water-extractable native SOM as a proxy of native bioavailable labile organic compounds from soils. Solid-phase extraction was performed on all samples to desalinate and concentrate the DOM solutions, according to Dittmar et al. (2008): briefly, extracts were diluted when necessary to 100 mg L^{-1} DOC with mass spectrometry grade water, and 25 mL of DOM solutions was processed through primed 100 mg Agilent PPL™ solid-phase extraction cartridges, resulting in maximum loadings of 2.5 mg C. The adsorbed OM was eluted with 1 mL of mass spectrometry grade methanol for FTICR-MS analysis, which was conducted at the College of Sciences Major Instrumentation Cluster at Old Dominion University. We recovered 71% of DOC after desalination and concentration

processing, which is higher than the average recovery (62%) reported previously (Dittmar et al. 2008).

The solid-phase extraction-processed samples, eluted in methanol, were diluted by a factor of 5 with LCMS grade methanol and water to give a final sample composition of 50:50 (v/v) methanol:water. Samples were continuously infused into an Apollo II electrospray (ESI) ion source of a Bruker Daltonics 12 T Apex Qe FTICR-MS operating in negative ion mode. Samples were introduced by a syringe pump providing an infusion rate of 120 μL h^{-1}. Ions (in the range of 200–1200 *m/z*) were accumulated in a hexapole for 0.5–1.0 s before being transferred to the ICR cell. Exactly 300 transients, collected with a 4 MWord time domain, were added for a total run time of approximately 30 min. The summed free induction decay signal was zero-filled once and Sine-Bell apodized prior to fast Fourier transformation and magnitude calculation using the Bruker Daltonics Data Analysis software, and the *m/z* width was set at 2 ppm for peak integration. Prior to data analysis, all samples were externally calibrated with a polyethylene glycol standard and internally calibrated with naturally present fatty acids and other compounds containing a CH_2 homologous series within the sample. Only *m/z* values with a signal to noise ratio (S/N) ≥5 were exported for formula assignment. All samples were analyzed with the same instrument parameters to ensure analytic consistency.

ESI FTICR-MS post-processing

A molecular formula-calculating MATLAB script developed by the Hatcher group at Old Dominion University was used to generate empirical formula matches for the resolved peaks using combinations of ^{12}C (8–50 atoms), H (8 ‒ 100 atoms), O (1-30 atoms), N (0 ‒ 5 atoms), S (0 ‒ 1 atoms), P (0 ‒ 1 atoms) present in unenriched, and ^{13}C (0 - 20) in ^{13}C enriched samples, as limiting atomic values. The resulting list was constrained to chemically feasible organic matter

formulae using the following criteria: $O/C \leqslant 1.2$, $H/C \leqslant 2.25$, $H/C \geqslant 0.3$, $N/C \leqslant 0.5$, $S/C \leqslant 0.2$, $P/C \leqslant 0.1$, $(S + P)/C \leqslant 0.2$, double-bond equivalence (DBE) $\geqslant 0$, and must be a whole number.

For all unenriched samples, the most appropriate molecular formula was selected using a hierarchy for determining the correct assignment based on the following: (1) Kendrick mass defect analysis, (2) least number of non-oxygen heteroatoms, and (3) lowest parts per million *m/z* deviation (<1 ppm). An iterative approach, designed by Rachel Sleighter, was used to assign molecular formulae in ^{13}C enriched samples, in which the maximum number of ^{12}C-containing formulae were assigned using the hierarchical approach detailed above, and from the remaining unassigned formulae, only ^{13}C-containing formulae in which a ^{13}C for ^{12}C replacement within previously-assigned ^{12}C-containing formulae occurred were assigned. This conservative approach, minimizing manual assignment, *m/z* error and heteroatom content, resulted in assignment of ~93% of spectral area. Following formulae assignment, only formulae present in at least two out of three analytical triplicates were deemed present.

Once assigned, formulae were classified into the appropriate van Krevelen space, which consisted of six discrete regions relying on the modified aromaticity index (AI_{mod}) calculation derived by Koch and Dittmar (2006): (1) polycyclic aromatic formulas (PCA; AImod> 0.66), (2) aromatic formulas ($0.66 \geqslant$ AImod> 0.50), (3) lignin/phenolic formulas (AImod $\leqslant 0.50$ and H/C < 1.5), (4) nitrogen-less aliphatic compounds, referred to as N- aliphatic ($2.0 > H/C \geqslant 1.5$ and N=0), (5) nitrogen-containing aliphatic compounds, referred to as N+ aliphatic ($2.0 > H/C \geqslant 1.5$ and N>0) and (6) carbohydrate-like compounds ($H/C \geqslant 2.0$ or $O/C \geqslant 0.9$). Nominal oxidation state of carbon (NOSC) was derived from molecular formulae using the following formulae from Riedel et al. (2012): $NOSC = 4 - [(4c + h - 3n - 2o - 2s)/c]$, where c, h, n, o, and s refer to the

stoichiometric numbers of carbon, hydrogen, nitrogen, oxygen, and sulfur atoms per formula, respectively.

Mössbauer Analysis

In order to isolate the added ^{57}Fe spikes from the underlying ^{57}Fe in the native soil, we performed a spectral subtraction at each temperature assuming the total spectral area at a given temperature was equivalent to the total ^{57}Fe in the sample. The relative intensities of the subtracted background (^{57}Fe signal in the native soil) only accounted for 8.8-18.5% of total ^{57}Fe (spike ^{57}Fe and native soil ^{57}Fe) in the system. Because each sample resulted in a different baseline count value and different variation in spectral width, we first normalized the native soil Fe spectra so that it had the same spectral area as the ^{57}Fe-enriched spectra and had the same baseline. Then, at each velocity value (mm/s), the normalized spectral intensity (counts per sec) of the native soil Fe spectra was subtracted from the ^{57}Fe-enriched spectra. The result was a new spectrum with the underlying soil native Fe subtracted.

Mössbauer spectral fitting was performed using the Voigt-based fitting method of Rancourt and Ping (1991) as implemented in the Recoil™ software. The relative abundance of each Fe site population (e.g., mineral phase) was extracted from the spectral fitting as a fraction of the total Fe spectral area. All errors in Mössbauer fitting parameters are two-standard deviation (2σ) errors, as calculated by Recoil™. Quantifying Fe phase abundance in this manner assumes equal Mössbauer recoilless fractions of all detected phases. This assumption is expected to be valid at cryogenic temperatures (Lalonde et al., 1998; Rancourt, 1998).

In Mössbauer spectra, each spectral component corresponds to one Fe-bearing solid phase or to a group of unresolved Fe-bearing solid phases. These components take the form of a doublet, sextet, octet (none resolved here) or a collapsed sextet — indicating a solid-phase near

its magnetic ordering temperature (T_N). Solid-phases well above (doublet) or below (sextet) their T_N will not exhibit any vertical (i.e., count axis) distance between the peak troughs and the baseline. When Fe solid-phases are near their T_N, they exhibit an intermediate shape between a doublet and full sextet, which fills the area between the upper baseline and the inverse troughs of the peaks. We approximate this by using a separate collapsed sextet component (i.e., a sextet with exceedingly large line widths and Bhf = 0 T).

Across the four collection temperatures we resolved five to seven distinct spectral components. Spectral components include: (1) an Fe^{III} quadrupole doublet corresponding to Fe^{III} in silicates, surface-complexed to solids, and in all Fe^{III}-(oxy)hydroxides that are superparamagnetic (SP) at the collection temperature; (2) a wide Fe^{II} quadrupole doublet that we attribute to paramagnetic ferrous in silicates or sorbed Fe^{II}; (3) a Fe^{III} sextet that has Bhf above 52.0 T and corresponds to hematite, (4) a broadened Fe^{III} sextet that has a negative quadrapole splitting and corresponds to phases best approximated by short-range-ordered (SRO) or nano-goethite; (5) a broadened Fe^{III} sextet that has a near zero quadrapole splitting and corresponds to phases best approximated by lepidocrocite; (6) a partially collapsed Fe^{III} 'sextet' due to a Fe^{III}-(oxy)hydroxide having its SP blocking temperature near the collection temperature; and (7) a partially magnetically ordered Fe^{II} phase evident only in the lowest collection temperature. In the 5 K spectra, if we assume all the Fe^{III}-oxyhydroxides have magnetically ordered, we can refine our component descriptions of the Fe^{III} quadrupole doublet to correspond to Fe^{III} in silicates and/or Fe-OM complexes; the Fe^{III} sextets (Ha-, Lp- and Gt-like) would represents phases with clear distinctive similarity to hematite, lepidocrocite and goethite, whereas the most disordered end-members of all Fe-oxide phases would be represented by the partially collapsed Fe^{III} 'sextet' attributed to Fe^{III}-(oxyhydr)oxides having their SP blocking temperatures near 5 K. Ferrous

phases form a doublet that is shifted to a higher velocity range than the Fe^{III} doublet. This ferrous doublet represents Fe^{II} in phyllosilicates and/or adsorbed to organic or mineral surfaces (Thompson et al. 2011). At low temperatures (5 K in our case), some ferrous populations can order into a collapsed octet characterized by strong asymmetry, which likely represents Fe^{II} sorbed onto a magnetically ordered Fe^{III} phase.

Fe^{III} (oxyhydr)oxides of lower crystallinity require lower measurement temperatures to magnetically order (and hence form a sextet) than Fe^{III} (oxyhydr)oxides of higher crystallinity. The area of Fe^{III} sextet increases as the measurement temperature decreases since Fe^{III} (oxyhydr)oxide phases of lower crystallinity—still represented by a doublet at a higher measurement temperature—are added to the respective sextet at the lower measurement temperature. The proportions of magnetically ordered Fe^{III} (oxyhydr)oxides binning to a certain crystallinity class were quantified by the area of the respective Fe^{III} (oxyhydr)oxide sextet measured at a certain temperature. Fe^{III} (oxyhydr)oxides that have not yet ordered at a specific measurement temperature appear as either a collapsed feature or as part of the Fe^{III} doublet in the Mössbauer spectrum.

Supplementary Notes

FTICR-MS results

Characterization of the molecular composition of ^{13}C-DOM and water-extractable native SOM using ultrahigh resolution mass spectrometry revealed two distinct populations of organic compounds (Supplementary Figure 5 and 6). Water-extractable native SOM was comprised of largely polycyclic aromatic (21.5%), lignin-derived/carboxyl-rich alicyclic molecules (49.1%) and aliphatic compounds (Supplementary Table 3), with mean population O/C, H/C and DBE values of 0.21±0.09, 1.21±0.35 and 13.73±7.51, respectively. In contrast, ^{13}C-DOM, derived from plant residues, was dominated by aliphatic formulae (76.3%) and lignin-derived/carboxyl-rich alicyclic molecules (23.0%), with mean population O/C, H/C and DBE values of 0.44±0.12, 1.60±0.22, and 6.31±3.04, respectively. Importantly, the 10.3% ^{13}C enrichment resulted in 2,023 ^{13}C-containing formulae contributing to almost 50% of spectral area detected in ^{13}C-DOM samples, and an identical chemical composition to ^{12}C-only formulae (Supplementary Figure 6 and Table 3), suggesting no compound-preferential incorporation of ^{13}C into plant compounds.

Mössbauer results

Solid-phase partitioning of the added ^{57}Fe following the initial 1-day anoxic period

When $^{57}Fe^{II}$ was added to soil slurries under anoxic conditions prior to exposure to O_2, sorption and electron transfer occurred. We distinguished these sorption products from products resulting from the oxidation event by characterizing the solid-phase ^{57}Fe following the initial 1-d anoxic reaction of the soil slurries with $^{57}Fe^{II}$. The Mössbauer spectra of the sorbed $^{57}Fe^{II}$ (corrected to exclude the signal from the native soil Fe, see methods) yielded strong sextets (Supplementary Figure 9), indicating electron transfer reactions occurred between the spiked

$^{57}Fe^{II}$ and native soil Fe^{III} atoms during the initial 1 day anoxic incubation. Most of the sorbed $^{57}Fe^{II}$ had been oxidized to nanogoethite (41.2% of the sorbed ^{57}Fe) and the most disordered Fe(III)-oxides (26.2% of the sorbed ^{57}Fe) (Supplementary Table 1). About 4% of sorbed $^{57}Fe^{II}$ was observed in the clay/OM Fe^{III} pool, suggesting a minor fraction of Fe electron transfer and atom exchange occurs between Fe^{II} and clay minerals or organic complexes. Examining the Mössbauer spectra at varying temperatures indicated that the amended ^{57}Fe selectively accumulated in the lower crystallinity portions of the Fe^{III}-oxide population, which displayed a magnetic ordering temperature below 35 K (Table 1; Supplementary Figure 9). Following the initial 1-day anoxic period, 29 – 32.6% of the sorbed $^{57}Fe^{II}$ remain un-oxidized, as revealed by Mössbauer spectra (Supplementary Figure 9 and Table 1). ^{13}C-DOM addition only slightly attenuated the electron transfer between the added $^{57}Fe^{II}$ and soil native Fe^{III} (Supplementary Figure 9 and Table 1).

Solid-phase partitioning of the amended ^{57}Fe during the anoxic phase of the fluctuating redox treatment

During the subsequent anoxic period following 1st oxidation, newly formed SRO Fe^{III} oxides ordered at 12 and 5K were preferentially reduced (Table 1; Supplementary Figure 7). A substantial proportion of the amended ^{57}Fe was reduced to Fe^{II} (7.5 – 36.2% of the added ^{57}Fe, Supplementary Table 1). Addition of ^{13}C-DOM and ^{57}Fe together yielded 28.7% more solid-phase $^{57}Fe^{II}$ than ^{57}Fe-addition only (Supplementary Figure 7 and Table 1). At the end of the anoxic phase, the lower crystallinity SRO Fe^{III-}oxides (those ordering at temperatures of 12 or 5 K in the Mössbauer spectra) were substantially decreased, while the more crystalline Fe^{III}-oxides ordering at 35 and 77 K exhibited only minor changes in spectral area (Table 1; Supplementary Figure 7).

Solid-phase partitioning of the amended ^{57}Fe during 2nd oxidation event of the fluctuating redox treatment

When soils were re-exposed to O_2 following anoxic incubation, a second redox fluctuation promoted Fe^{II} oxidation and its subsequent precipitation of Fe^{III} (oxyhdr)oxides (Supplementary Figure 8 and Table 1). When ^{57}Fe was added alone, Fe^{II} re-oxidation resulted in a mineral composition with similar crystallinity to what was formed during the first oxidation (Figure 3; Table1; Supplementary Table 1). However, when ^{13}C-DOM and ^{57}Fe were added together, the Fe^{III} oxides formed via Fe^{II} re-oxidation displayed a higher crystallinity ratio (12K/5K) than those formed during the first oxidation (Table 1; compare supplementary Figure 3 and Figure 8).

Supplementary Figures

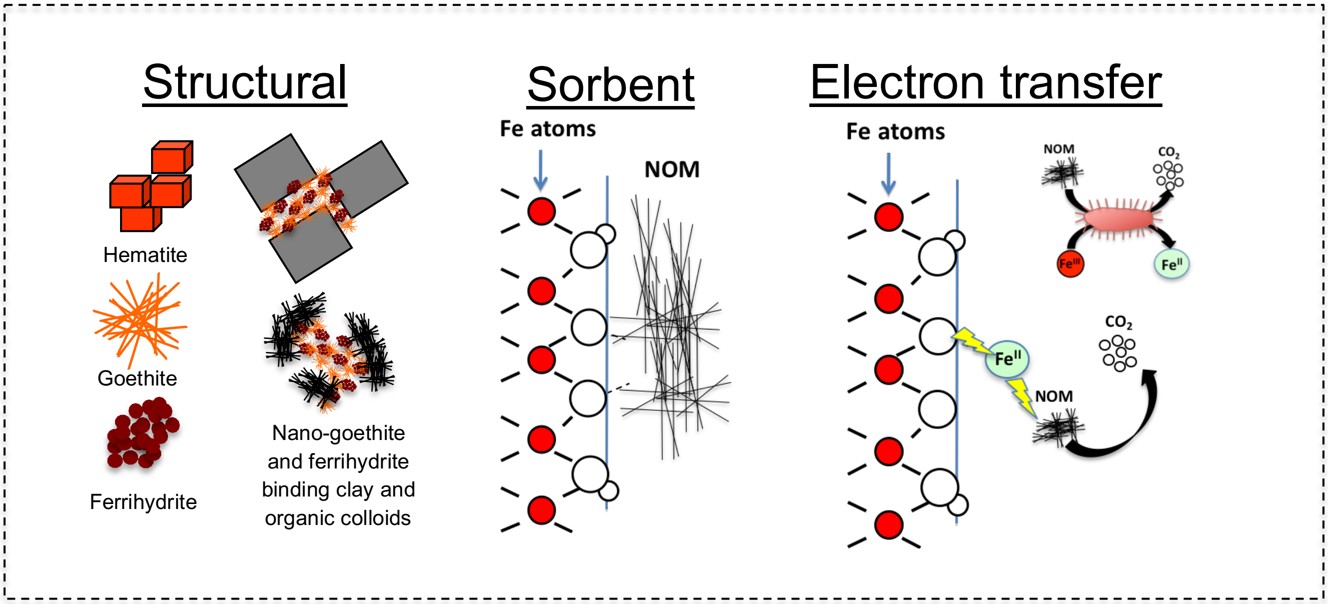

Supplementary Figure 1: The functional roles of Fe in soil C cycling.

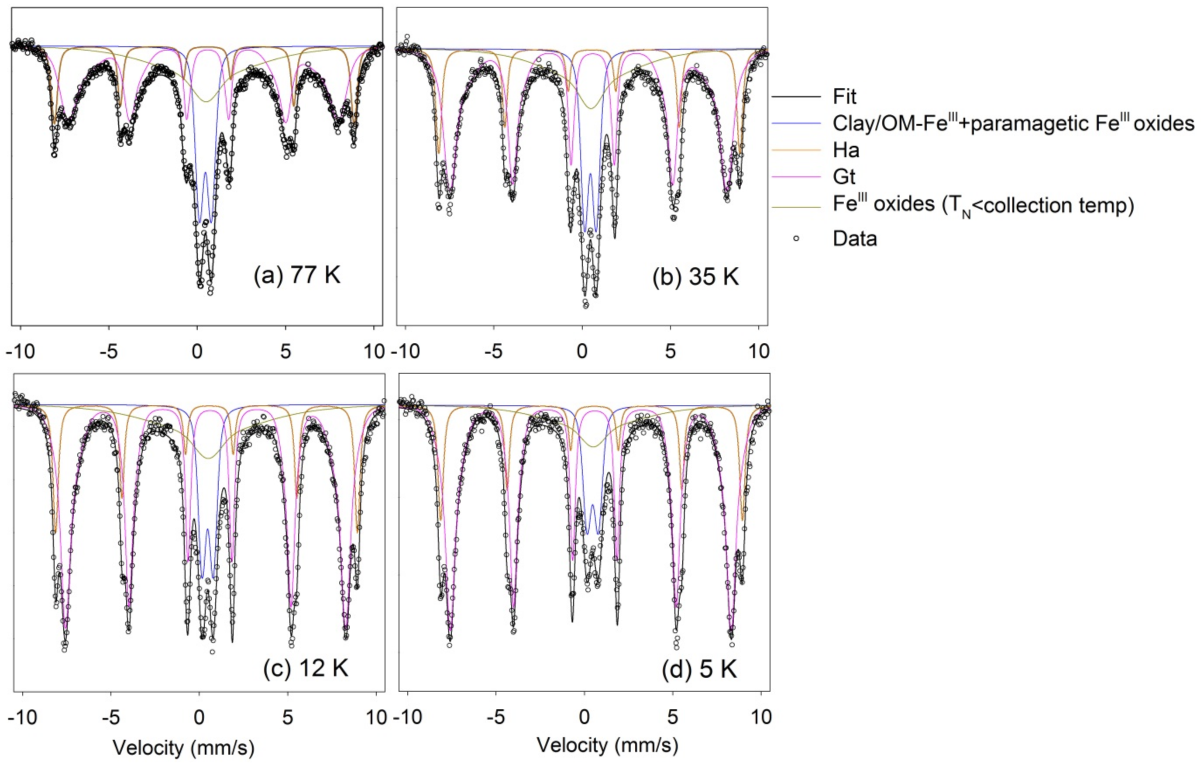

Supplementary Figure 2: ^{57}Fe Mössbauer spectra of the initial unreacted soil at 77 K, 35 K, 12K and 5 K. In each spectrum, the black line is the total calculated fit, through the discrete data points. The resolved spectral components and assignments are: (1) Fe^{III} in aluminosilicates and in organic complexes (+ paramagnetic Fe^{III} (oxyhydr)oxides) (blue line, labeled as "clay/OM-Fe^{III} + paramagnetic Fe^{III} oxides"); (2) Fe^{III} in hematite (red line, labeled as "Ha"); (3) Fe^{III} in goethite (purple line, labeled as "Gt"); and (4) Fe^{III} (oxyhydr)oxides near their blocking temperature (dark yellow line, labeled as "Fe^{III} oxides (T_N<collection temp)"). Note that Fe^{III}(oxyhydr)oxides near the blocking temperature-5 K ("Fe^{III} oxides (T_N<5K)") represent the most disordered forms. The detailed fitting parameters are presented in Supplementary Table 4.

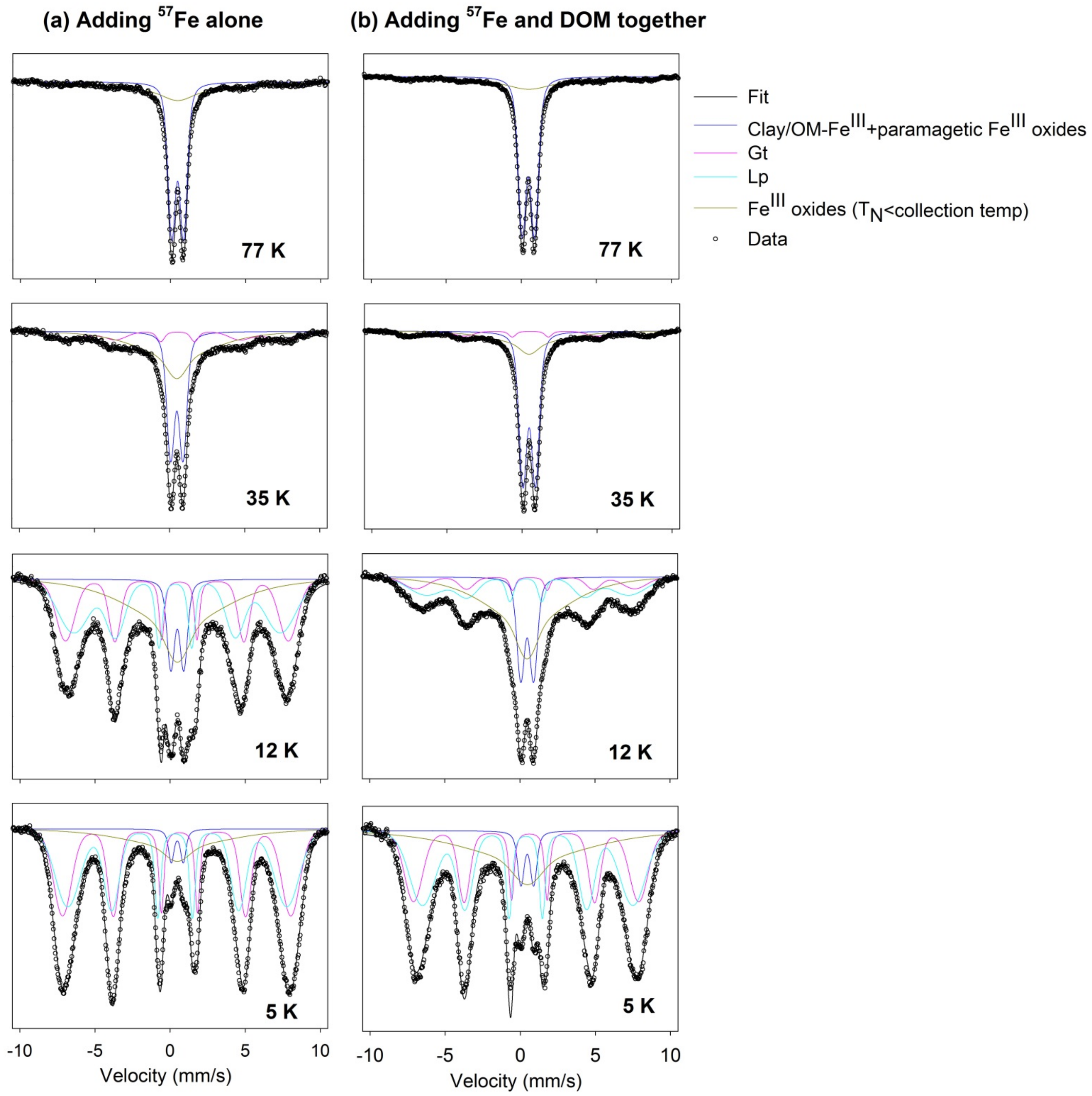

Supplementary Figure 3: ^{57}Fe Mössbauer spectra (77K, 35K, 12K and 5K) of the amended ^{57}Fe (corrected to exclude the signal from the native soil Fe) at the end of first oxic period (day 6). In each spectrum, the black line is the total calculated fit, through the discrete data points. The resolved spectral components and assignments are: (1) Fe^{III} in silicates and in organic complexes (+ paramagnetic Fe^{III} oxyhydroxides) (blue line, labeled as "clay/OM-Fe^{III} + paramagnetic Fe^{III} oxides"); (2) Fe^{III} in goethite (purple line, labeled as "Gt"); (3) Fe^{III} in lepidocrocite (cyan line, labeled as "Lp"); and (4) Fe^{III} (oxyhydr)oxides near their blocking temperature (dark yellow line, labeled as "Fe^{III} oxides (T_N<collection temp)"). Note that Fe^{III}(oxyhydr)oxides near the blocking temperature-5 K ("Fe^{III} oxides (T_N<5K)") represent the most disordered forms. The detailed fitting parameters are presented in Supplementary Table 7 and Table 8.

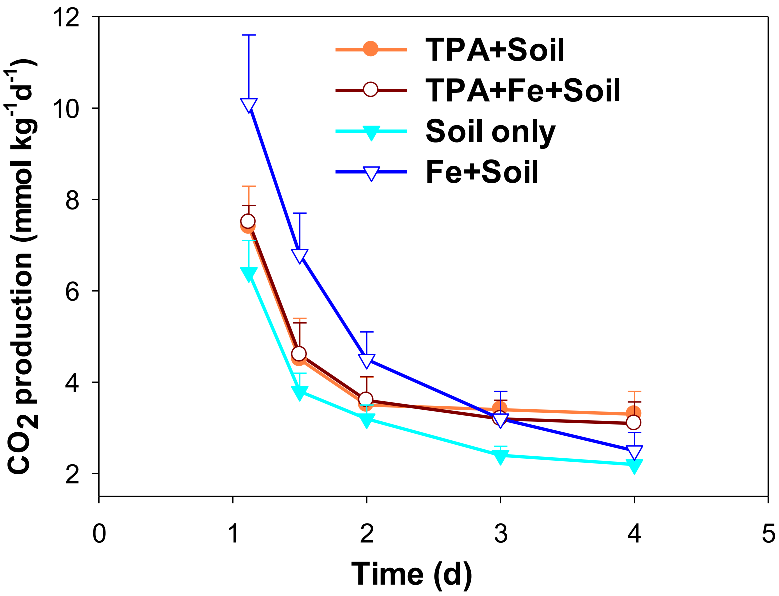

Supplementary Figure 4: The impact of TPA addition on soil CO_2 production under oxic condition. The error bars indicates s.e.m. (n=3).

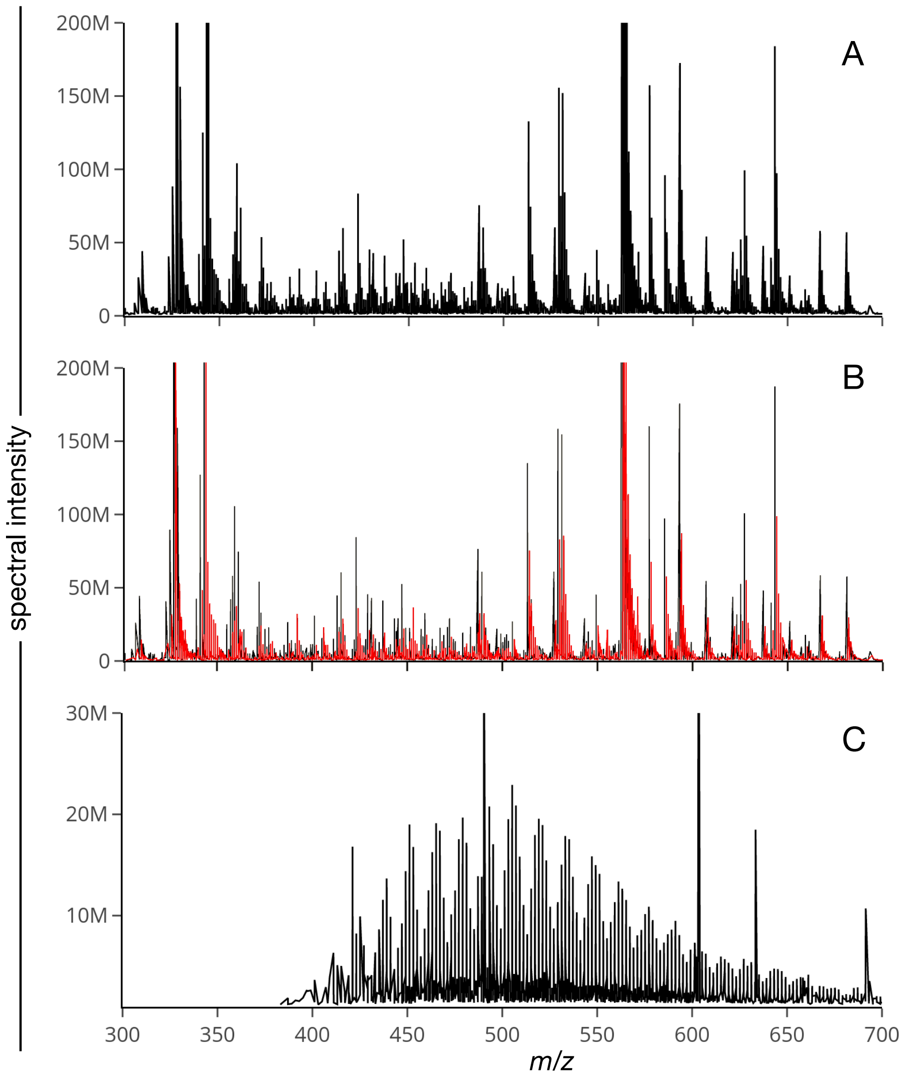

Supplementary Figure 5: FTICR-MS spectra of all DOM formulae (A), ^{13}C-DOM enriched formulae highlighted in red (B), and water-extractable native SOM (C).

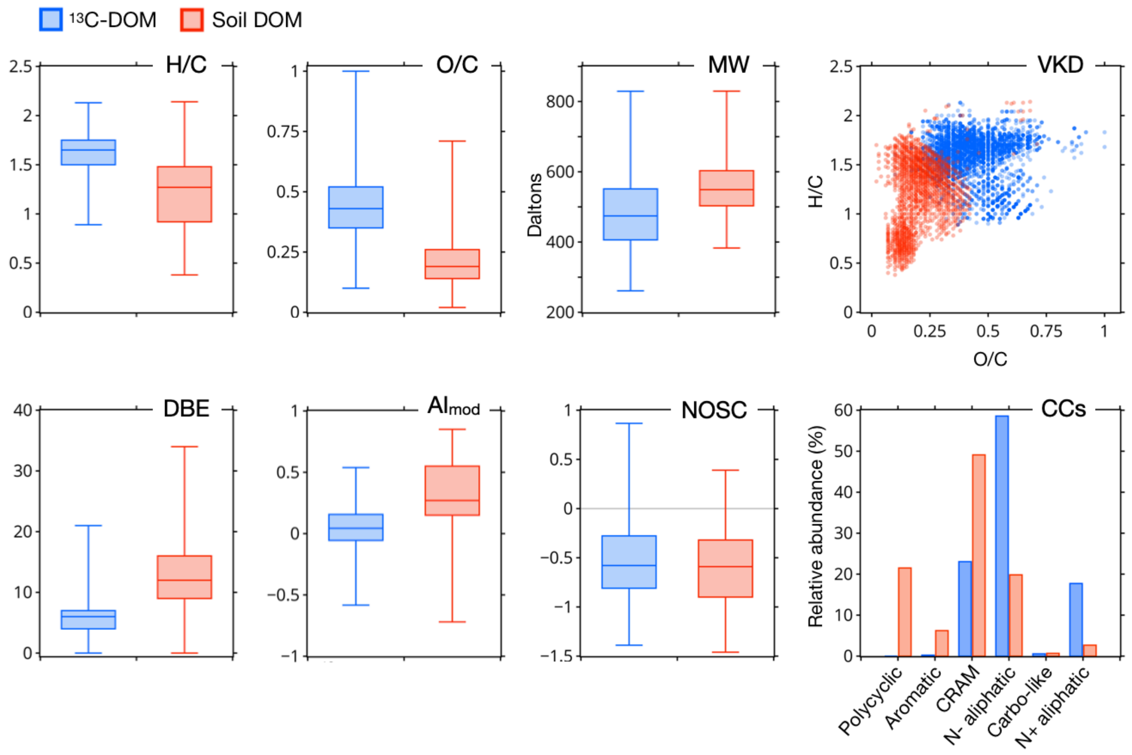

Supplementary Figure 6:The molecular formulae parameters of ^{13}C-DOM and water-extractable native SOM derived from FTICR-MS analysis: H/C and O/C ratios, molecular weight (MW), van Krevelen space diagram (VKD), double bond equivalence (DBE), aromaticity index (AI_{mod}, Koch & Dittmar (2006)), nominal oxidation state of carbon (NOSC, Riedel et al. (2012)), and relative abundance across six assigned compound classes (CCs). Across boxplots, the boxes enclose the interquartile range, the whiskers indicate minimum and maximum values, and horizontal bars dissecting the boxes represent the median values. Details on FTICR-MS analysis, post-processing, and associated calculations can be found in the above Supplementary methods.

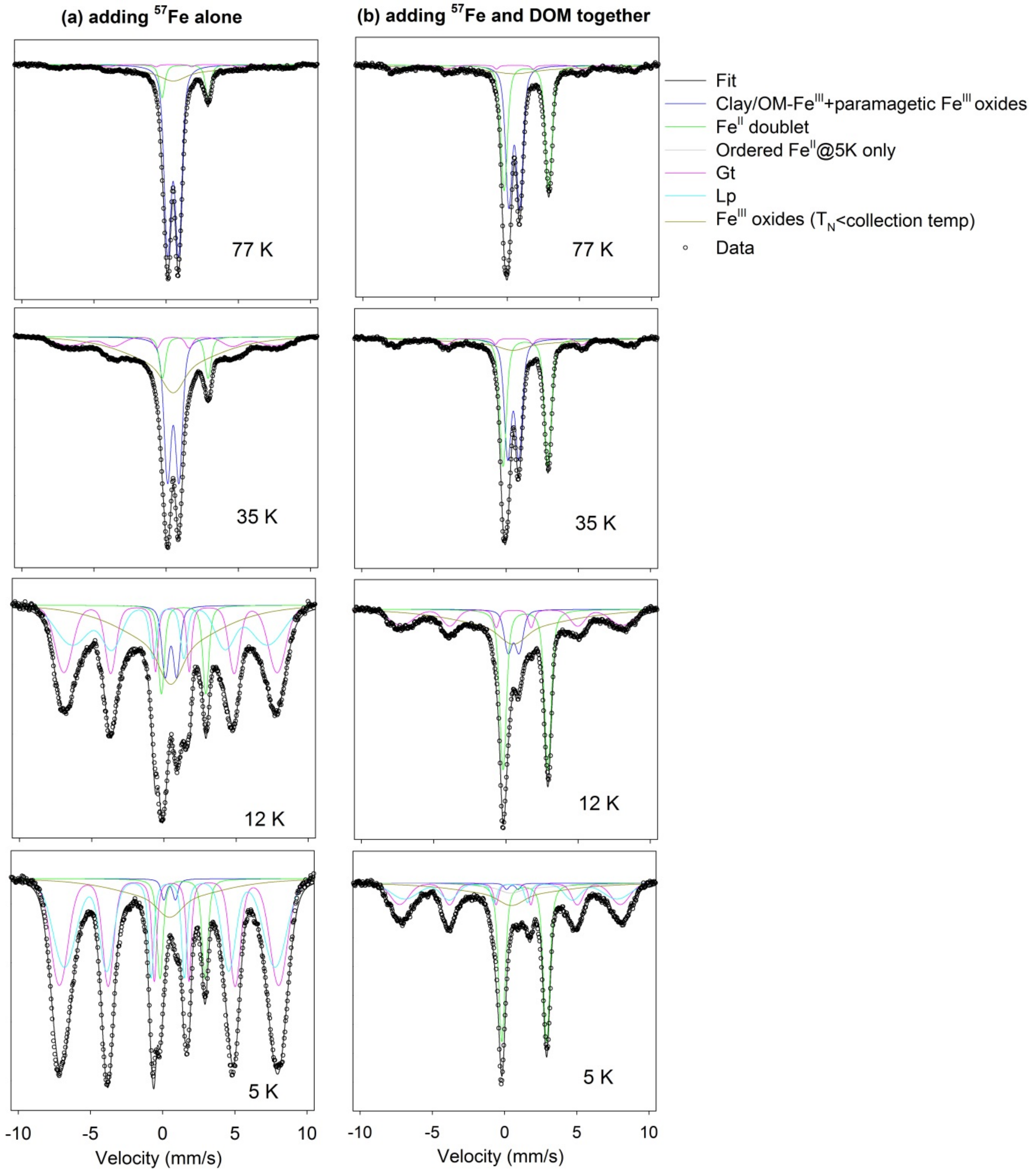

Supplementary Figure 7: ^{57}Fe Mössbauer spectra (77K, 35K, 12K and 5K) of the amended ^{57}Fe (corrected to exclude the signal from the native soil Fe) at the end of subsequent anoxic period following the first oxidation event (day 17). In each spectrum, the black line is the total calculated fit, through the discrete data points. The resolved spectral components and oxyhydroxides) (blue line, labeled as "clay/OM-Fe^{III} + paramagnetic Fe^{III} oxides"); (2) Fe^{II} in clays or sorbed (green line, labeled as "Fe^{II} doublet"); (3) magnetically order Fe^{II} at 5K (gray line, labeled as "ordered Fe^{II} @5K Only"); (4) Fe^{III} in goethite (purple line, labeled as "Gt"); (5) Fe^{III} in lepidocrocite (cyan line, labeled as "Lp"); and (6) Fe^{III} (oxyhydr)oxides near their blocking temperature (dark yellow line, labeled as "Fe^{III} oxides (T_N<collection temp)"). Note that Fe^{III}(oxyhydr)oxides near the blocking temperature-5 K ("Fe^{III} oxides (T_N<5K)") represent the most disordered forms. The detailed fitting parameters are presented in Supplementary Table 9 and Table 10.

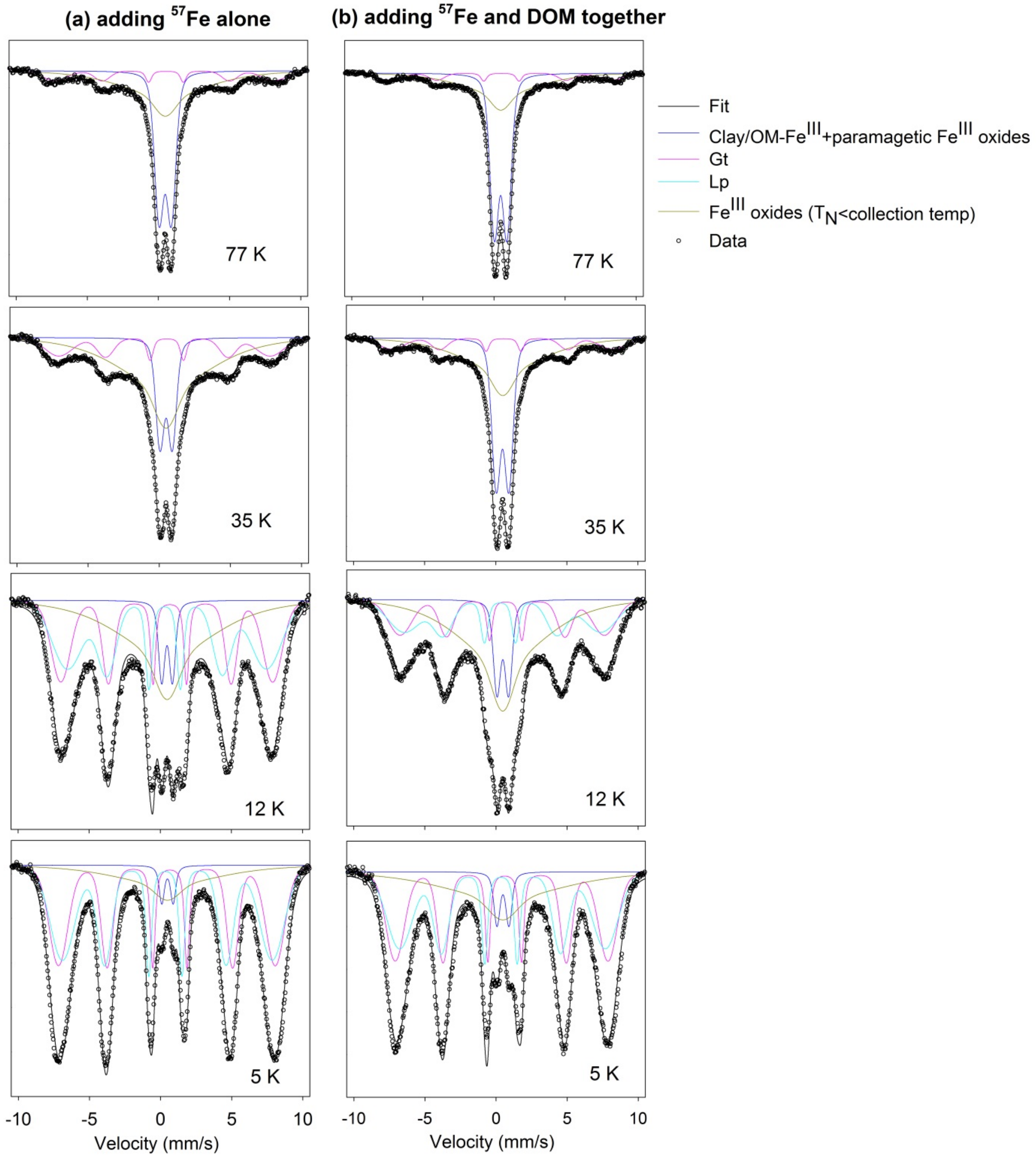

Supplementary Figure 8: ^{57}Fe Mössbauer spectra (77K, 35K, 12K and 5K) of the amended ^{57}Fe (corrected to exclude the signal from the native soil Fe) at the end of secibd oxic period (day 22). In each spectrum, the black line is the total calculated fit, through the discrete data points. The resolved spectral components and assignments are: (1) Fe^{III} in silicates and in organic complexes (+ paramagnetic Fe^{III} oxyhydroxides) (blue line, labeled as "clay/OM-Fe^{III} + paramagnetic Fe^{III} oxides"); (2) Fe^{III} in goethite (purple line, labeled as "Gt"); (3) Fe^{III} in lepidocrocite (cyan line, labeled as "Lp"); and (4) Fe^{III} (oxyhydr)oxides near their blocking temperature (dark yellow line, labeled as "Fe^{III} oxides (T_N<collection temp)"). Note that Fe^{III}(oxyhydr)oxides near the blocking temperature-5 K ("Fe^{III} oxides (T_N<5K)") represent the most disordered forms. The detailed fitting parameters are presented in Supplementary Table 11 and Table 12.

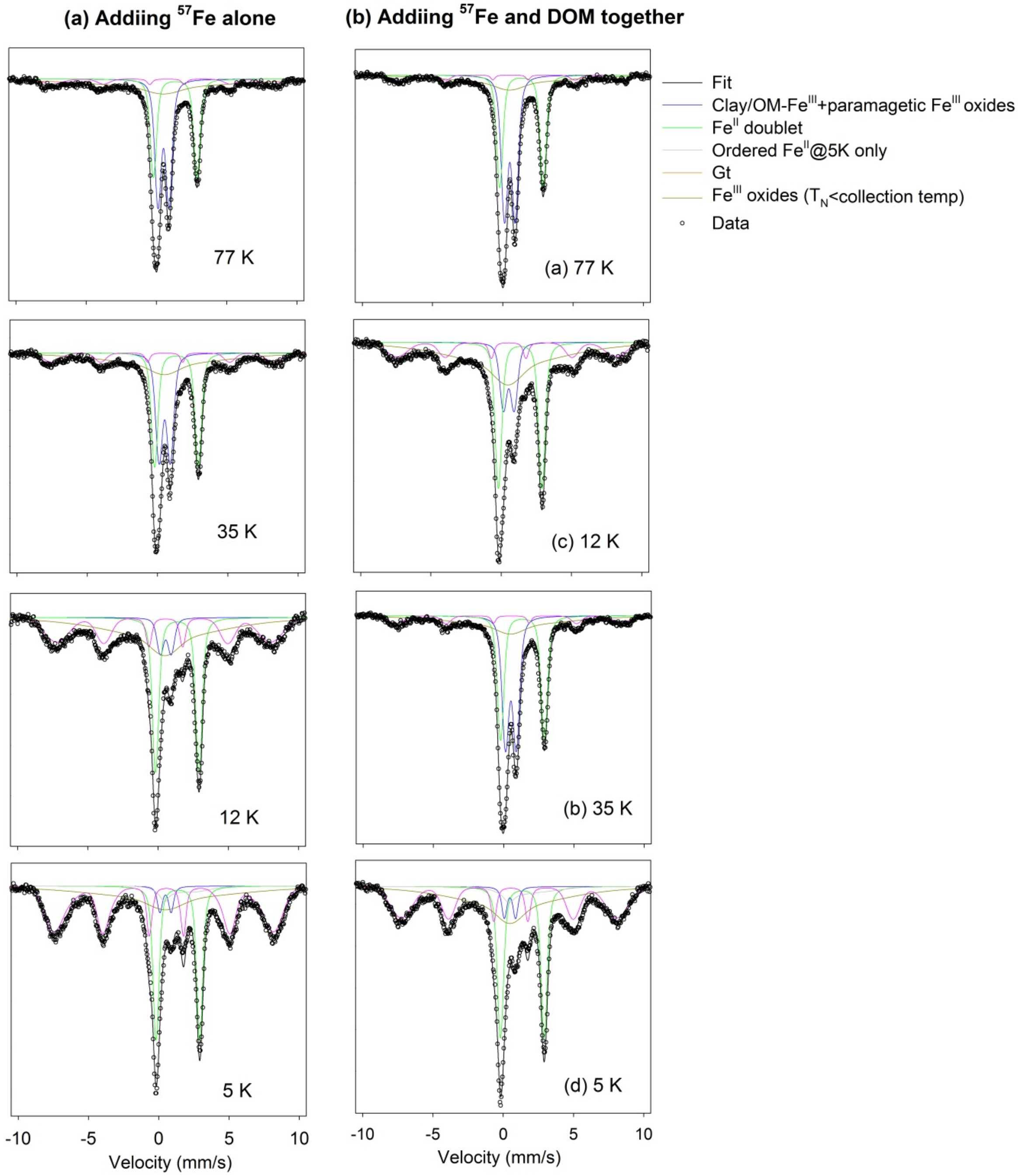

Supplementary Figure 9: ^{57}Fe Mössbauer spectra (77K, 35K, 12K and 5K) of the amended ^{57}Fe (corrected to exclude the signal from the native soil Fe) in the solid phase after the initial sorptive reaction with soils for 1 d under anoxic conditions prior to oxidation. In each spectrum, the black line is the total calculated fit, through the discrete data points. The resolved spectral components and assignments are: (1) Fe^{III} in aluminosilicates and in organic complexes (+ paramagnetic Fe^{III} (oxyhydr)oxides) (blue line, labeled as "clay/OM-Fe^{III} + paramagnetic Fe^{III} oxides"); (2) Fe^{II} in clays or sorbed (green line, labeled as "Fe^{II} doublet"); (3) ordered Fe^{II} at 5K only (gray line); (4) Fe^{III} in goethite (purple line, labeled as "Gt"); and (5) Fe^{III} (oxyhydr)oxides near their blocking temperature (dark yellow line, labeled as "Fe^{III} oxides (T_N<collection temp)"). Note that Fe^{III} (oxyhydr)oxides near the blocking temperature-5 K ("Fe^{III} oxides (T_N<5K)") represent the most disordered forms. The detailed fitting parameters are presented in Supplementary Table 5 and Table 6.

Supplementary Tables

Supplementary Table 1: Partition of the amended ^{57}Fe in the solid phase as calculated from respective Mössbauer spectra (corrected to exclude the signal from the native soil Fe).

**$^{57}Fe^{II}$-only addition**	**mmol kg^{-1}**				**% of total Fe**			
	Prior to oxic (1 d)	End of 1st oxic (6 d)	End of anoxic (17 d)	End of 2nd oxic (22 d)	Prior to oxic (1 d)	End of 1st oxic (6 d)	End of anoxic (17 d)	End of 2nd oxic (22 d)
Organic/silicated Fe^{III}	1.0 (0.1)	1.6 (0.1)	0.7 (0.1)	1.6 (0.1)	3.6 (0.4)	2.4 (0.2)	1.2 (0.2)	2.4 (0.1)
Fe^{II}	8.7 (0.2)	nd	4.4 (0.1)	nd	29.0 (0.6)	nd	7.5 (0.1)	nd
nano-geothite	12.1 (0.3)	26.1 (1.0)	22.5 (0.3)	26.4 (0.7)	41.2 (1.0)	38.5 (1.4)	38.2 (0.5)	38.9 (1.0)
lepidocrocite	nd	31.1 (1.0)	24.1 (0.3)	31.7 (0.7)	nd	45.8 (1.4)	40.9 (0.5)	46.7 (1.0)
Most SRO Fe^{III} oxides	7.7 (0.4)	9.0 (0.7)	7.2 (0.4)	8.3 (0.4)	26.2 (1.3)	13.3 (1.0)	12.2 (0.6)	12.2 (0.6)

**$^{57}Fe^{II}$ and ^{13}C-DOM addition**	**mmol kg^{-1}**				**% of total Fe**			
	Prior to oxic (1 d)	End of 1st oxic (6 d)	End of anoxic (17 d)	End of 2nd oxic (22 d)	Prior to oxic (1 d)	End of 1st oxic (6 d)	End of anoxic (17 d)	End of 2nd oxic (22 d)
Organic/silicated Fe^{III}	1.4 (0.1)	2.6 (0.2)	0.4 (0.2)	2.2 (0.1)	3.9 (0.4)	3.8 (0.3)	1.0 (0.4)	3.3 (0.2)
Fe^{II}	11.8 (0.4)	nd	15.6 (0.4)	nd	32.6 (1.0)	nd	36.2 (1.0)	nd
nano-geothite	12.5 (0.7)	23.5 (1.1)	10.6 (0.6)	30.0 (0.8)	34.7 (1.9)	34.6 (1.7)	24.6 (1.4)	41.2 (1.2)
lepidocrocite	nd	26.8 (1.1)	7.5 (0.7)	24.8 (0.8)	nd	39.4 (1.7)	17.4 (1.7)	36.6 (1.2)
Most SRO Fe^{III} oxides	10.4 (0.7)	15.1 (1.0)	9.0 (0.7)	12.8 (0.7)	28.8 (2.0)	22.2 (1.4)	20.8 (1.5)	18.9 (1.1)

Numbers in parenthesis represent S.E.M. (n=3).

Supplementary Table 2: Primed CO_2 loss for all treatments.

Substrate treatment	Time	Redox treatment	Primed CO_2 (mmol C kg^{-1})
Fe^{II}-added soils	1-6 d	1st-oxic/ fluctuating	3.0
		static oxic	2.8
	6-17 d	anoxic/ fluctuating	2.7
		static oxic	0.4
	17-22 d	2nd-oxic/fluctuating	-0.8
		static oxic	0.1
	Sum (1-22 d)	fluctuating	4.9
		static oxic	3.5
DOM-added soils	1-6 d	1st-oxic/ fluctuating	8.7
		static oxic	9.0
	6-17 d	anoxic/ fluctuating	0.1
		static oxic	7.4
	17-22 d	2nd-oxic/fluctuating	-0.3
		static oxic	2.7
	Sum (1-22 d)	fluctuating	8.5
		static oxic	19.2
DOM- and Fe^{II}-added soils	1-6 d	1st-oxic/ fluctuating	4.3
		static oxic	5.1
	6-17 d	anoxic/ fluctuating	2.3
		static oxic	4.1
	17-22 d	2nd-oxic/fluctuating	-1.6
		static oxic	1.0
	Sum (1-22 d)	fluctuating	5.0
		static oxic	10.3

Supplementary Table 3: Mean formulae parameters (DBE: double-bond equivalence; MW: molecular weight; NOSC: nominal oxidation of carbon; AI_{mod}: modified aromaticity index) and compound relative abundances, namely polycyclic aromatic (PCA), aromatic carboxyl-rich alicyclic molecules (CRAM), nitrogen-containing (N+) and –less (N-) aliphatic, and carbohydrate-like compounds. Parameters are described for water-extractable native SOM, and plant-derived ^{13}C-DOM across all formulae, ^{12}C-containing formulae, and ^{13}C-containing populations.

		Mean formulae population parameters						*Relative abundance of compound classes*					
		**H/C**	**O/C**	**DBE**	**MW**	**NOSC**	**AI_{mod}[1]**	**PCA**	**Aromatic**	**Lignin-derived/CRAM**	**N- aliphatic**	**Carbo-like**	**N+ aliphatic**
**Water-extractable SOM**		0.21	1.21	13.73	554.1	-0.61	0.33	21.50%	6.22%	49.10%	19.83%	0.68%	2.67%
**^{13}C-DOM**	***All formulae***	0.44	1.60	6.31	484.4	-0.54	0.05	0.00%	0.24%	23.05%	58.57%	0.56%	17.72%
	^{12}C only	0.43	1.59	6.49	481.8	-0.53	0.07	0.00%	0.40%	25.90%	53.04%	0.94%	21.95%
	^{13}C only	0.45	1.61	6.13	487.0	-0.54	0.04	0.00%	0.10%	20.66%	65.25%	0.20%	13.84%

[1] A modified aromaticity index (AI_{mod}) calculation was derived from Koch and Dittmar (2006).

Supplementary Table 4: Mössbauer parameters and site populations for the initial unreacted soil at 77 K, 35 K, 12 K and 5 K. The spectra are presented in Supplementary Figure 5.

Temperature	Phase	Spectral Area	CS	QS	Δ or H	σ	*Red-X²*
		%	mm/s	mm/s	mm/s or T	mm/s or T	
77K	Q-Fe^{III}	19.04(57)	0.4607(59)	n/a	0.6738(89)	0.330(13)	1.16
	Ha-like	14.5(10)	0.4748(65)	-0.0887(65)	52.595(65)	0.61(11)	
	Gt-like	43.2(12)	0.4675(98)	-0.1239(95)	45.39(14)	5.138(38)	
	Fe^{III} oxides (T_N<77K)	23.3(10)	0.50*	0*	0*	24.99(21)	
35K	Q-Fe^{III}	15.54(54)	0.4707(62)	n/a	0.6553(96)	0.3035(60)	1.40
	Ha-like	15.10(90)	0.4816(55)	-0.0890(55)	52.855(55)	0.496(96)	
	Gt-like	51.7(12)	0.4742(52)	-0.1260(52)	47.4908(67)	4.2376(21)	
	Fe^{III} oxides (T_N<35K)	17.7(12)	0.50*	0*	0*	21.1 (29)	
12K	Q-Fe^{III}	12.61(48)	0.4678(74)	n/a	0.632(11)	0.302(15)	1.30
	Ha-like	15.2(11)	0.4819(50)	-0.0818(52)	52.952(46)	0.42(10)	
	Gt-like	55.4(13)	0.4745(35)	-0.1242(35)	48.7425(43)	2.8989(18)	
	Fe^{III} oxides (T_N<12K)	16.7(12)	0.50*	0*	0*	26.3(37)	
5K	Q-Fe^{III}	10.99(60)	0.470(12)	n/a	0.636(18)	0.33*	1.16
	Ha-like	15.5(16)	0.4850(64)	-0.0852(66)	52.962(59)	0.86(14)	
	Gt-like	60.9(16)	0.4738(39)	-0.1173(39)	49.1833(45)	2.73(21)	
	Most disordered Fe^{III}-oxides	12.6(12)	0.50*	0*	0*	22.3(42)	

CS, center shift; QS, quadrupole shift; Δ, quadrupole splitting; H, hyperfine field shift; σ, standard deviation of Δ or H; *Red-X²*, goodness of fit. Errors in parentheses are 2 s.d. displayed in concise form indicating the error in the last digit [e.g., 7.1(12) is equivalent to 7.1 ± 1.2]. * indicates values that were fixed during the fitting process.

Supplementary Table 5: Mössbauer parameters and site populations for the amended ^{57}Fe (corrected to exclude the signal from the native soil Fe) in the solid phase after sorptive reaction with soils for 1 d under anoxic conditions, prior to oxidation in the treatment of adding ^{57}Fe alone. The spectra are presented in Supplementary Figure 6a.

Temperature	Phase	Spectral Area	CS	QS	Δ or H	σ	*Red-X²*
		%	mm/s	mm/s	mm/s or T	mm/s or T	
77K	Q-Fe^{III}	36.19(85)	0.4973(28)	n/a	0.7845(45)	0.3168(65)	2.28
	Q-Fe^{II}	28.99(69)	1.3300(31)	n/a	3.0982(61)	0.3151(85)	
	Gt-like	8.1(11)	0.503(52)	-0.124(51)	48.35(47)	4.21(61)	
	Fe^{III} oxides (T_N<77K)	26.7(14)	0.50*	0*	0*	43.0(43)	
35K	Q-Fe^{III}	29.26(69)	0.5068(41)	n/a	0.7979(64)	0.3609(91)	2.00
	Q-Fe^{II}	30.38(68)	1.3461(31)	n/a	3.1134(62)	0.3297(86)	
	Gt-like	11.35(99)	0.455(30)	-0.0882(30)	49.17(27)	3.49(38)	
	Fe^{III} oxides (T_N<35K)	29.0(12)	0.50*	0*	0*	36.1 (32)	
12K	Q-Fe^{III}	6.79(52)	0.542(17)	n/a	0.772(26)	0.342(37)	2.54
	Q-Fe^{II}	29.21(87)	1.3620(32)	n/a	3.0941(64)	0.3285(87)	
	Gt-like	29.2(14)	0.467(18)	-0.086(18)	48.65(11)	1.84(14)	
	Fe^{III} oxides (T_N<12K)	34.8(16)	0.50*	0*	0*	34.7(31)	
5K	Q-Fe^{III}	3.64(43)	0.4883(87)	n/a	0.798(27)	0.357(22)	2.76
	Q-Fe^{II}	24.84(57)	1.3628(31)	n/a	3.1050(61)	0.2944(80)	
	Gt-like	41.2(10)	0.4534(66)	-0.0883(40)	47.1097(62)	4.8432(82)	
	Most disordered Fe^{III}-oxides	26.2(13)	0.50*	0*	0*	42.834(97)	
	Ordered Fe(II)	4.21(72)	1.40*	1.0*	0*	7.02(22)	

CS, center shift; QS, quadrupole shift; Δ, quadrupole splitting; H, hyperfine field shift; σ, standard deviation of Δ or H; *Red-X²*, goodness of fit. Errors in parentheses are 2 s.d. displayed in concise form indicating the error in the last digit [e.g., 7.1(12) is equivalent to 7.1 ± 1.2]. * indicates values that were fixed during the fitting process.

Supplementary Table 6: Mössbauer parameters and site populations for the amended ^{57}Fe (corrected to exclude the signal from the native soil Fe) in the solid phase after sorptive reaction with soils for 1 d under anoxic conditions, prior to oxidation in the treatment of adding ^{57}Fe and ^{13}C-DOM together. The spectra are presented in Supplementary Figure 6b.

Temperature	Phase	Spectral Area	CS	QS	Δ or H	σ	*Red-X^2*
		%	mm/s	mm/s	mm/s or T	mm/s or T	
77K	Q-Fe^{III}	41.07(73)	0.5036(25)	n/a	0.7614(39)	0.3298(57)	2.20
	Q-Fe^{II}	32.03(58)	1.3286(26)	n/a	3.0918(52)	0.3077(72)	
	Gt-like	6.20(82)	0.500(42)	-0.078(42)	49.21(37)	2.88(50)	
	Fe^{III} oxides (T_N<77K)	20.7(11)	0.48*	0*	0*	33.2(35)	
35K	Q-Fe^{III}	35.54(58)	0.5159(27)	n/a	0.7707(41)	0.3552(60)	2.86
	Q-Fe^{II}	33.24(52)	1.3433(23)	n/a	3.1099(45)	0.3286(62)	
	Gt-like	8.68(72)	0.466(22)	-0.0882(36)	49.72(24)	2.99(33)	
	Fe^{III} oxides (T_N<35K)	22.53(91)	0.50*	0*	0*	32.9 (28)	
12K	Q-Fe^{III}	13.90(52)	0.5430(83)	n/a	0.769(12)	0.376(18)	2.22
	Q-Fe^{II}	32.45(70)	1.3558(29)	n/a	3.1001(58	0.3375(79)	
	Gt-like	16.0(11)	0.454(18)	-0.080(12)	48.31(22)	4.32(33)	
	Fe^{III} oxides (T_N<12K)	38.6(12)	0.50*	0*	0*	34.3(22)	
5K	Q-Fe^{III}	3.91(36)	0.4863(77)	n/a	0.796(29)	0.207(20)	2.44
	Q-Fe^{II}	26.79(72)	1.3615(30)	n/a	3.1103(59)	0.2985(78)	
	Gt-like	34.7(19)	0.483(12)	-0.083(12)	47.52(12)	4.47(17)	
	Most disordered Fe^{III}-oxides	28.8(20)	0.50*	0*	0*	32.0(29)	
	Ordered Fe(II)	5.8(10)	1.40*	1.0*	0*	10*	

CS, center shift; QS, quadrupole shift; Δ, quadrupole splitting; H, hyperfine field shift; σ, standard deviation of Δ or H; *Red-X^2*, goodness of fit. Errors in parentheses are 2 s.d. displayed in concise form indicating the error in the last digit [e.g., 7.1(12) is equivalent to 7.1 ± 1.2]. * indicates values that were fixed during the fitting process.

Supplementary Table 7: Mössbauer parameters and site populations for the amended ^{57}Fe (corrected to exclude the signal from the native soil Fe) in the solid phase at the end of first oxic phase (day 6) in the treatment of adding ^{57}Fe alone. The spectra are presented in Supplementary Figure 7a.

Temperature	Phase	Spectral Area	CS	QS	Δ or H	σ	*Red-X^2*
		%	mm/s	mm/s	mm/s or T	mm/s or T	
77K	Q-Fe^{III}	65.42(75)	0.4734(22)	n/a	0.7857(34)	0.3650(50)	2.03
	Fe^{III} oxides (T_N<77K)	34.58(75)	0.50*	0*	0*	31.9(20)	
35K	Q-Fe^{III}	38.7(16)	0.4820(36)	n/a	0.8245(55)	0.3768(95)	0.89
	Gt-like	18.5(24)	0.471(54)	-0.091(50)	44.23(72)	7.13(71)	
	Fe^{III} oxides (T_N<35K)	42.8(19)	0.50*	0*	0*	21.6 (24)	
12K	Q-Fe^{III}	8.53(57)	0.4738(95)	n/a	0.861(15)	0.365(24)	1.70
	Gt-like	25.8(19)	0.511(12)	-0.092(12)	46.01(15)	3.43(16)	
	Lp-like	36.6(22)	0.423(18)	0.017(17)	42.69(28)	6.13(25)	
	Fe^{III} oxides (T_N<12K)	29.0(18)	0.50*	0*	0*	27.0(31)	
5K	Q-Fe^{III}	2.36(20)	0.4812(97)	n/a	0.778(27)	0.257(32)	3.14
	Gt-like	38.5(14)	0.5329(73)	-0.0916(76)	47.149(69)	3.312(72)	
	Lp-like	45.8(14)	0.4082(94)	0.0304(96)	45.261(95)	4.731(96)	
	Most disordered Fe^{III}-oxides	13.30(97)	0.50*	0*	0*	28.7(33)	

CS, center shift; QS, quadrupole shift; Δ, quadrupole splitting; H, hyperfine field shift; σ, standard deviation of Δ or H; *Red-X^2*, goodness of fit. Errors in parentheses are 2 s.d. displayed in concise form indicating the error in the last digit [e.g., 7.1(12) is equivalent to 7.1 ± 1.2]. * indicates values that were fixed during the fitting process.

Supplementary Table 8: Mössbauer parameters and site populations for the amended ^{57}Fe (corrected to exclude the signal from the native soil Fe) in the solid phase at the end of first oxic phase (day 6) in the treatment of adding ^{57}Fe and ^{13}C-DOM together. The spectra are presented in Supplementary Figure 7b.

Temperature	Phase	Spectral Area	CS	QS	Δ or H	σ	*Red-X²*
		%	mm/s	mm/s	mm/s or T	mm/s or T	
77K	Q-Fe^{III}	70.40(46)	0.4761(11)	n/a	0.8221(18)	0.3811(26)	3.45
	Fe^{III} oxides (T_N<77K)	29.60(46)	0.50*	0*	0*	39.7(18)	
35K	Q-Fe^{III}	62.03(80)	0.4838(14)	n/a	0.8319(23)	0.3856(36)	3.09
	Gt-like	13.14(81)	0.490(41)	-0.087(39)	47.24(43)	6.22(42)	
	Fe^{III} oxides (T_N<35K)	24.83(68)	0.50*	0*	0*	19.2 (13)	
12K	Q-Fe^{III}	18.1(13)	0.4858(49)	n/a	0.8572(76)	0.382(15)	0.84
	Gt-like	11.4(14)	0.513(41)	-0.126(51)	45.23(59)	4.26(59)	
	Lp-like	25.3(14)	0.459(34)	0.037(38)	41.72(57)	7.00(57)	
	Fe^{III} oxides (T_N<12K)	45.1(31)	0.50*	0*	0*	23.1(25)	
5K	Q-Fe^{III}	3.79(33)	0.470(13)	n/a	0.851(22)	0.286(34)	2.72
	Gt-like	34.6(17)	0.5003(33)	-0.1088(42)	46.5138(55)	3.40(11)	
	Lp-like	39.4(17)	0.4333(83)	0.0344(94)	43.46(13)	4.99(11)	
	Most disordered Fe^{III}-oxides	22.2(14)	0.50*	0*	0*	33.9(27)	

CS, center shift; QS, quadrupole shift; Δ, quadrupole splitting; H, hyperfine field shift; σ, standard deviation of Δ or H; *Red-X²*, goodness of fit. Errors in parentheses are 2 s.d. displayed in concise form indicating the error in the last digit [e.g., 7.1(12) is equivalent to 7.1 ± 1.2]. * indicates values that were fixed during the fitting process.

Supplementary Table 9: Mössbauer parameters and site populations for the amended ^{57}Fe (corrected to exclude the signal from the native soil Fe) in the solid phase at the end of subsequent anoxic period following the first oxidation event (day 17) in the treatment of adding ^{57}Fe alone. The spectra are presented in Supplementary Figure 8a.

Temperature	Phase	Spectral Area	CS	QS	Δ or H	σ	*Red-X^2*
		%	mm/s	mm/s	mm/s or T	mm/s or T	
77K	Q-Fe^{III}	64.73(83)	0.4778(13)	n/a	0.7560(19)	0.3340(27)	3.10
	Q-Fe^{II}	9.77(27)	1.3007(55)	n/a	3.181(11)	0.211(16)	
	Gt-like	3.49(74)	0.436(67)	-0.095(67)	48.51(59)	3.21(82)	
	Fe^{III} oxides (T_N<77K)	22.01(78)	0.50*	0*	0*	26.8(21)	
35K	Q-Fe^{III}	32.39(52)	0.4888(13)	n/a	0.7914(19)	0.3504(32)	3.43
	Q-Fe^{II}	8.52(17)	1.3209(34)	n/a	3.1639(67)	0.2663(93)	
	Gt-like	15.26(92)	0.438(17)	-0.0772(16)	43.99(19)	6.41(26)	
	Fe^{III} oxides (T_N<35K)	43.82(76)	0.50*	0*	0*	24.39(97)	
12K	Q-Fe^{III}	6.49(41)	0.491(10)	n/a	0.791(16)	0.335(25)	2.60
	Q-Fe^{II}	7.59(31)	1.3650(63)	n/a	3.079(13)	0.273(18)	
	Gt-like	28.2(14)	0.5240(79)	-0.0881(79)	45.802(94)	3.34(11)	
	Lp-like	29.7(17)	0.405(18)	0.0381(16)	41.89(29)	6.62(27)	
	Fe^{III} oxides (T_N<12K)	28.0(16)	0.50*	0*	0*	26.7(27)	
5K	Q-Fe^{III}	1.25(17)	0.4801(77)	n/a	0.801(22)	0.237(20)	3.55
	Q-Fe^{II}	7.48(14)	1.3757(31)	n/a	3.1098(32)	0.2650(60)	
	Gt-like	38.19(46)	0.5234(28)	-0.0913(28)	47.0997(74)	3.3452(82)	
	Lp-like	40.85(50)	0.4287(15)	0.0421(58)	45.2586(22)	4.49759(55)	
	Most disordered Fe^{III}-oxides	12.22(55)	0.50*	0*	0*	27.8(20)	

CS, center shift; QS, quadrupole shift; Δ, quadrupole splitting; H, hyperfine field shift; σ, standard deviation of Δ or H; *Red-X^2*, goodness of fit. Errors in parentheses are 2 s.d. displayed in concise form indicating the error in the last digit [e.g., 7.1(12) is equivalent to 7.1 ± 1.2]. * indicates values that were fixed during the fitting process.

Supplementary Table 10: Mössbauer parameters and site populations for the amended ^{57}Fe (corrected to exclude the signal from the native soil Fe) in the solid phase at the end of subsequent anoxic period following the first oxidation event (day 17) in the treatment of adding ^{57}Fe and ^{13}C-DOM together. The spectra are presented in Supplementary Figure 8b.

Temperature	Phase	Spectral Area	CS	QS	Δ or H	σ	*Red-X²*
		%	mm/s	mm/s	mm/s or T	mm/s or T	
77K	Q-Fe^{III}	40.84(72)	0.4945(22)	n/a	0.7598(36)	0.3121(51)	3.00
	Q-Fe^{II}	37.71(67)	1.3361(21)	n/a	3.0898(42)	0.3097(59)	
	Gt-like	5.24(73)	0.472(48)	-0.086(48)	48.20(43)	2.95(56)	
	Fe^{III} oxides (T_N<77K)	16.2(12)	0.50*	0*	0*	44.9(62)	
35K	Q-Fe^{III}	36.97(51)	0.5035(23)	n/a	0.7735(36)	0.3339(54)	3.87
	Q-Fe^{II}	40.18(51)	1.3470(18)	n/a	3.1109(35)	0.3179(50)	
	Gt-like	7.93(59)	0.483(24)	-0.087(24)	48.32(21)	2.63(28)	
	Fe^{III} oxides (T_N<35K)	14.92(79)	0.50*	0*	0*	28.3(31)	
12K	Q-Fe^{III}	10.28(42)	0.542(10)	n/a	0.759(14)	0.407(22)	3.98
	Q-Fe^{II}	35.20(73)	1.3599(19)	n/a	3.1024(38)	0.3195(51)	
	Gt-like	21.0(10)	0.479(17)	-0.088(16)	47.49(15)	4.72(24)	
	Fe^{III} oxides (T_N<12K)	33.5(11)	0.50*	0*	0*	31.2(22)	
5K	Q-Fe^{III}	0.99(29)	0.5422(17)	n/a	0.801(22)	0.237(20)	3.44
	Q-Fe^{II}	32.0(10)	1.3662(22)	n/a	3.0960(42)	0.3132(57)	
	Gt-like	24.6(14)	0.5001(26)	-0.1216(27)	47.4256(54)	3.5352(20)	
	Lp-like	17.4(17)	0.4372(16)	0.0121(68)	45.6234(26)	5.3528(26)	
	Most disordered Fe^{III}-oxides	20.8(15)	0.50*	0*	0*	28.4(30)	
	Ordered Fe(II)	4.21(21)	1.4*	1.0*	0*	20.0(36)	

CS, center shift; QS, quadrupole shift; Δ, quadrupole splitting; H, hyperfine field shift; σ, standard deviation of Δ or H; *Red-X²*, goodness of fit. Errors in parentheses are 2 s.d. displayed in concise form indicating the error in the last digit [e.g., 7.1(12) is equivalent to 7.1 ± 1.2]. * indicates values that were fixed during the fitting process.

Supplementary Table 11: Mössbauer parameters and site populations for the amended ^{57}Fe (corrected to exclude the signal from the native soil Fe) in the solid phase at the end of second-oxic phase (day 22) in the treatment of adding ^{57}Fe alone. The spectra are presented in Supplementary Figure 9a.

Temperature	Phase	Spectral Area	CS	QS	Δ or H	σ	*Red-X²*
		%	mm/s	mm/s	mm/s or T	mm/s or T	
77K	Q-Fe^{III}	44.8(12)	0.4737(36)	n/a	0.8634(50)	0.5095(84)	0.95
	Gt-like	14.7(17)	0.477(36)	-0.071(50)	47.94(35)	5.10(53)	
	Fe^{III} oxides (T_N<77K)	40.5(14)	0.50*	0*	0*	26.4(23)	
35K	Q-Fe^{III}	21.09(58)	0.4829(44)	n/a	0.8630(62)	0.459(10)	1.67
	Gt-like	20.8(13)	0.451(19)	-0.089(18)	46.11(18)	5.62(29)	
	Fe^{III} oxides (T_N<35K)	58.1(11)	0.50*	0*	0*	27.9 (13)	
12K	Q-Fe^{III}	5.32(24)	0.4740(75)	n/a	0.754(12)	0.291(19)	3.15
	Gt-like	28.54(91)	0.5196(76)	-0.1076(80)	46.193(77)	3.782(87)	
	Lp-like	37.2(10)	0.407(12)	0.052(11)	43.38(13)	6.32(13)	
	Fe^{III} oxides (T_N<12K)	28.98(98)	0.50*	0*	0*	29.4(18)	
5K	Q-Fe^{III}	2.43(14)	0.480(23)	n/a	0.801(12)	0.216(24)	3.85
	Gt-like	38.85(99)	0.5226(73)	-0.1049(76)	47.260(55)	3.717(60)	
	Lp-like	46.7(10)	0.4333(83)	0.0376(89)	45.746(67)	4.949(73)	
	Most disordered Fe^{III}-oxides	12.24(57)	0.50*	0*	0*	30.9(29)	

CS, center shift; QS, quadrupole shift; Δ, quadrupole splitting; H, hyperfine field shift; σ, standard deviation of Δ or H; *Red-X*^{2}, goodness of fit. Errors in parentheses are 2 s.d. displayed in concise form indicating the error in the last digit [e.g., 7.1(12) is equivalent to 7.1 ± 1.2]. * indicates values that were fixed during the fitting process.

Supplementary References

1. Keiluweit, M., Wanzek,T., Kleber, M., Nico, P. & Fendorf, S. Anaerobic microsites have unaccounted role in soil carbon stabilization. *Nat. Commun.* 8, 1171 (2018).
2. Dittmar, T., Koch, B., Hertkorn, N. & Kattner, G. A simple and efficient method for the solid-phase extraction of dissolved organic matter (SPE-DOM) from seawater. *Limnol. Oceanogr. : Methods* 6 (6), 230–235 (2008).
3. Koch, B. P. & Dittmar, T. From mass to structure: An aromaticity index for high-resolution mass data of natural organic matter. *Rapid Commun. Mass Spectrom.* 20 (5), 926–932 (2006).
4. Riedel, T., Biester, H. & Dittmar, T. Molecular Fractionation of Dissolved Organic Matter with Metal Salts. *Environ. Sci. Technol.* 46 (8), 4419–4426 (2012).
5. Rancourt, D. G. & Ping J. Voigt-based methods for arbitrary-shape static hyperfine parameter distributions in Mössbauer spectroscopy. *Nucl. Instrum. Meth. Phys. Res. Sect. B* 58, 85–97 (1991).
6. Rancourt, D. G. Mössbauer spectroscopy in clay science. *Hyperfine Interact.* 117, 3–38 (1998).
7. Lalonde, A. E., Rancourt, D. G. & Ping, J. Y. Accuracy of ferric/ferrous determinations in micas: A comparison of Mössbauer spectroscopy and the Pratt and Wilson wet-chemical methods. *Hyperfine Interact.* 117, 175–204 (1998).
8. Thompson, A., Chadwick, O. A., Rancourt, D. G. & Chorover, J. Iron solid-phase differentiation along a redox gradient in basaltic soils. *Geochim. Cosmochim. Acta* 75(1), 119–133 (2011).
